# Alternative Oxidase Dependent Respiration Leads to an Increased Mitochondrial Content in Two Long-Lived Mutants of the Ageing Model *Podospora anserina*

**DOI:** 10.1371/journal.pone.0016620

**Published:** 2011-01-27

**Authors:** Christian Q. Scheckhuber, Koen Houthoofd, Andrea C. Weil, Alexandra Werner, Annemie De Vreese, Jacques R. Vanfleteren, Heinz D. Osiewacz

**Affiliations:** 1 Faculty for Biosciences, Molecular Developmental Biology, Cluster of Excellence Macromolecular Complexes, Johann Wolfgang Goethe University, Frankfurt, Germany; 2 Department of Biology and Center for Molecular Phylogeny and Evolution, Ghent University, Ghent, Belgium; University Medical Center Groningen, The Netherlands

## Abstract

The retrograde response constitutes an important signalling pathway from mitochondria to the nucleus which induces several genes to allow compensation of mitochondrial impairments. In the filamentous ascomycete *Podospora anserina*, an example for such a response is the induction of a nuclear-encoded and iron-dependent alternative oxidase (AOX) occurring when cytochrome-c oxidase (COX) dependent respiration is affected. Several long-lived mutants are known which predominantly or exclusively respire via AOX. Here we show that two AOX-utilising mutants, grisea and *PaCox17*::ble, are able to compensate partially for lowered OXPHOS efficiency resulting from AOX-dependent respiration by increasing mitochondrial content. At the physiological level this is demonstrated by an elevated oxygen consumption and increased heat production. However, in the two mutants, ATP levels do not reach WT levels. Interestingly, mutant *PaCox17*::ble is characterized by a highly increased release of the reactive oxygen species (ROS) hydrogen peroxide. Both grisea and *PaCox17*::ble contain elevated levels of mitochondrial proteins involved in quality control, i. e. LON protease and the molecular chaperone HSP60. Taken together, our work demonstrates that AOX-dependent respiration in two mutants of the ageing model *P. anserina* is linked to a novel mechanism involved in the retrograde response pathway, mitochondrial biogenesis, which might also play an important role for cellular maintenance in other organisms.

## Introduction

The ascomycete *Podospora anserina* is a filamentous fungus extensively used as an experimental model organism to study the molecular basis of organismal ageing [1]–[5]. During the last decades it was demonstrated that there is an environmental as well as a genetic basis of ageing and lifespan control. A hallmark of ageing in *P. anserina* WT strains is the reorganisation of the mitochondrial DNA (mtDNA) accompanied by mitochondrial dysfunction [6]–[8]. Stabilisation of the mtDNA results in lifespan extension. More recent investigations revealed that the type of respiration has an important impact on the ageing of cultures [9]–[15]. Normally, strains respire via a cytochrome-c oxidase (COX, complex IV) dependent respiratory chain. However, when this pathway is impaired for different reasons, a compensating mechanism, termed the retrograde response, is induced and leads to the expression of a gene coding for an alternative oxidase (AOX). This enzyme receives electrons from the ubiquinone pool and reduces oxygen directly, hence by-passing the electron flux through cytochrome-c reductase (complex III) and COX. Electron transfer via AOX was found to result in reduced reactive oxygen species (ROS) levels in isolated mitochondria and protoplasts, respectively [11], [14]. A long-lived *P. anserina* mutant respiring predominantly via AOX is grisea [16] which is a loss-of-function mutant of the *Grisea* gene that encodes a copper regulated transcription factor [17]. Since this transcription factor controls the expression of the high-affinity transporter PaCTR3, copper uptake in the mutant is restricted to a low affinity uptake system and results in cellular copper depletion [10], [18], [19]. Because copper is needed as a cofactor for COX activity, COX-depending respiration is impaired and alternative respiration is induced. Similarly, a deletion of the gene *PaCox17* encoding a mitochondrial chaperone delivering copper to a subunit of COX results in respiration via AOX and a pronounced lifespan extension [13]. Although long-lived when cultivated on cornmeal agar, both grisea and *PaCox17*::ble are characterized by severe physiological defects, like strongly decreased growth rate and reduced female fertility (mutant grisea) or female sterility (*PaCox17*::ble).

Due to the fact that the switch from the standard to the alternative type of respiration affects the proton motive force at the inner mitochondrial membrane, consequences on the efficiency of both ATP generation and ROS production rates are expected. We therefore measured different metabolic parameters including ATP levels, oxygen consumption, and heat production as well as hydrogen peroxide production, ROS scavenging capacity and protein levels of mitochondrial quality control components in living mycelia or homogenates from the wild type (WT) strain and compared these data with those of the two long-lived mutants grisea and *PaCox17*::ble which are predominantly (grisea) or exclusively (*PaCox17*::ble) respiring via the alternative pathway. Finally, measurements of mitochondrial content of the aforementioned strains experimentally address the question whether alternative respiration leads to changes in mitochondrial biogenesis.

## Methods

### Strains and media

In this study, WT ‘s’ [3], mutant grisea [16] and mutant *PaCox17*::ble [13] were analysed. Juvenile cultures were derived from mononucleate ascospores incubated on germination medium as previously described [19]. Pieces of mycelium were subsequently transferred onto cornmeal agar plates and cultivated at 27°C. After 8 d of growth, 20 pieces of the front of the mycelium were grown on a fresh cornmeal agar plate. After two days of growth, the mycelium was scraped off the plate and transferred to Erlenmeyer flasks containing liquid complete medium and shaken at 27°C. Three days later, the mycelium was harvested by filtering through gaze. 100 mg aliquots were stored at −80°C for determination of the different parameters. Living mycelium was used for the measurement of oxygen consumption and heat production. Protoplasts for the measurement of mitochondrial content were prepared as described previously [13].

### Metabolic measurements

Metabolic output was measured by applying oxygen consumption and heat production measurements, respectively. Consumption of dissolved oxygen by 20–50 mg of suspended mycelium in 1 ml liquid complete medium (CM) was monitored polarographically using Clark electrodes (Strathkelvin, North Lanarkshire, UK), at 27°C. Heat production by 20–50 mg of mycelium in 1 ml CM medium was measured by microcalorimetry in the ‘Thermal Activity Monitor’ (Thermometric, Parthenia St. Northridge, CA) at 27°C.

ATP levels were measured on flash frozen mycelium samples. We used the luciferin-luciferase assay kit (ATP Bioluminescence Assay Kit CLS II, Roche Diagnostics, Mannheim, Germany) adapted for use in a microtiter plate format as described previously [20]. This assay is based on the reaction: luciferin + ATP + O_2_ → oxyluciferin + AMP + pyrophosphate + CO_2_ + light. The frozen mycelium samples (100 mg) were taken from the −80°C freezer and immediately submersed in a boiling water bath for 25 minutes to destroy ATPase activity and to allow diffusion of ATP out of the mycelium. After 15 minutes of boiling, the samples were smashed with glass beads (Mini-Beadbeater, Merlin Diagnostic Systems, Breda, The Netherlands) for two minutes. Dilutions were made using HPLC grade water (salts interfere with the assay) and the assay was performed according to the manufacturer’s instructions.

### Analysis of mitochondrial morphology

*P. anserina* mycelia were grown on glass slides that have a central depression. This depression was filled with a 1:1 mixture of cornmeal agar and 1% agarose for two days in a wet chamber at 27°C. The mycelium was covered with 1 µM Mitotracker Green FM (Invitrogen, Carlsbad, CA). After 10 minutes of staining the samples at 27°C in the wet chamber, mitochondria were visualized using a fluorescence microscope equipped with appropriate excitation and emission filters (DM LB, Leica, Wetzlar, Germany).

### Quantitative determination of mitochondrial content

Mitochondrial content was quantified by applying the 10-*n*-nonyl acridine orange (NAO) method [21] to *P. anserina* protoplasts. 10^7^ protoplasts were stained in 1 ml 1 µM NAO in TPS buffer (5 mM Na_2_HPO_4_ · 2 H_2_O, 45 mM KH_2_PO_4_, 0.58 M sucrose, pH 5.5). After 10 min incubation at room temperature in the dark the sample was centrifuged (10 min, 15000 g). The protoplasts were washed twice in 1 ml TPS before they were resuspended in 200 µl TPS. NAO fluorescence of the protoplast suspension was subsequently measured in a multiplate reader (Safire2, Tecan, Salzburg, Austria) (excitation: 495 nm, emission: 519 nm). As a loading control, protein content was determined by the method of Bradford.

MtDNA levels as a marker of mitochondrial quantity were determined by PCR. Total DNA extracts (10 ng/reaction) were used as templates. Oligonucleotides binding in the mtDNA gene encoding the large ribosomal subunit, *PaLsu* (mtDNA_Q1f: 5′-GGGTACGACTGTTCGTCG-3′, mtDNA_Q1r: 5′-TTGGGTATACAACAGTACCCC-3′), were used in the reaction to analyse the amount of mitochondrial genomes. Amplification of the nuclear *PaGpd* gene [22] was performed to determine the amount of nuclear genomes (oligonucleotides Gpd_Q1f: 5′-ATCATCCCCAGCAGCACC-3′ and Gpd_Q1r: 5′-CACACGTCTGCTGTAGCC-3′ were used). Amplification products were separated on 1% agarose gels, stained with ethidiumbromide and quantified using ImageJ (http://imagej.nih.gov/ij/index.html).

PaPORIN levels in total protein extracts were also analysed as a marker of mitochondrial quantity by SDS-PAGE and Western blot analysis.

### Isolation of mitochondria

Mitochondria of *P. anserina* cultures were isolated by differential centrifugation as described previously [14].

### Isolation of total proteins

Total proteins were isolated according to the protocol published in [23].

### Isolation of total DNA

*P. anserina* DNA (i. e., genomic DNA and mtDNA) isolation was performed according to a previously published protocol [24].

### SDS-PAGE and Western blot analysis

80 µg of total protein or 100 µg of mitochondrial protein was incubated at 95°C for 10 min in loading buffer (0.1 M TRIS [pH 6.8], 6% SDS, 6% glycerol, 0.6 M β-mercaptoethanol, 0.08% bromophenolblue) and was subsequently separated by using 12% SDS-PAGE. After electrophoretic separation, proteins were transferred to a PVDF membrane (Immobilon-FL, Millipore, Schwalbach, Germany) using an electro-blotting system (Bio-Rad, Munich, Germany). Membranes were incubated in blocking buffer (Li-Cor, Lincoln, NE, USA) for 1 h at RT and subsequently probed with a polyclonal *P. anserina* PaLON protease antibody (Anti-PaLON) (1∶1500, overnight, 4°C), polyclonal *P. anserina* PaPRX1 (mitochondrial peroxiredoxin) antibody (Anti-PaPRX1) (1∶2000, overnight, 4°C), polyclonal *P. anserina* PaIAP (i-AAA protease) antibody (Anti-PaIAP) (1∶5000, overnight, 4°C), polyclonal *P. anserina* PaCLPP (CLP protease subunit) antibody (Anti-PaCLPP) (1∶400, overnight, 4°C) and a monoclonal HSP60 (mouse) antibody (Anti-HSP60) (1∶4000, overnight, 4°C) from Biomol Stressgen, Hamburg, Germany. Incubation with a polyclonal antibody against PaPORIN (Anti-PaPOR) (1∶5000, overnight, 4°C) was performed as loading control for mitochondrial protein preparations or to determine mitochondrial content in total protein extracts. Labelling was detected with IRDye 800 conjugated goat-anti-rabbit antibody or IRDye 680 conjugated goat-anti-mouse antibody (1∶10000, 1 h, RT) and scanning the blots with an Odyssey infrared scanner (Li-Cor, Lincoln, NE, USA). For densitrometric analysis of signal intensities the software package supplied with the Odyssey scanner was used according to the developer's instructions.

### Hydrogen peroxide production measurements

Qualitative determination of hydrogen peroxide release from mycelia was performed by monitoring oxidation of diaminobenzidine (DAB, Sigma Aldrich, St. Louis, MO) according to previously published protocols [25], [26].

### SOD and catalase activity

The activities of SOD and catalase were measured in homogenates that were prepared by bead-beating 100 mg frozen mycelium samples for 90 seconds after adding 300 µl of 50 mM Na/K-phosphate buffer (pH 7.0). SOD activity was measured by an assay based on the inhibition of superoxide-induced lucigenin chemiluminescence by SOD [27]. Aliquots of 6.7 µl were taken from a homogenate dilution series and added in duplicate to the wells of a microtiter plate. Next, 20 µl aliquots of xanthine oxidase (XO) reagent (XO diluted in double distilled water such that the blank reaction containing 6.7 µl water, 20 µl XO dilution and 174 µl reaction mixture yielded approx. 1.2×10^5^ counts/s) and 174 µl of reaction mixture (5.2 ml 0.1 M glycine, 1 mM EDTA, adjusted to pH 9.0 with NaOH, 10 ml 0.108 mM xanthine, 2.1 ml 1 mM lucigenin, 1.2 ml water for a total of 18.5 ml) was added quickly by using a multichannel pipette. Luminescence was measured for 0.1 s during the time span required for 25 consecutive plate measurements at 25°C using a Victor^2^ Multilabel Counter (Perkin Elmer, Waltham, MA). One unit of SOD activity is defined as the amount of SOD able to reduce the luminescence intensity by 50%. The homogenate fraction (dilution) reducing luminescence by 50% was derived mathematically from plots of the luminescence intensities measured as a function of the homogenate fraction [28] The sensitivity of this assay is superior to the standard cytochrome c assay [29], [30] and the numerical values of SOD activity are not comparable.

Catalase activity was assayed at 25°C according to the method of [31], adapted for use in microtiter plate format. Briefly 6.9 µl sample volumes were added to the wells of a 96-well flat bottom UV transparent microtiter plate (UV-Star, Greiner, Frickenhausen, Germany). The reaction was started by adding 200 µl substrate (11.4 mM hydrogen peroxide in 50 mM Na_2_HPO_4_ · KH_2_PO_4_ (Sorensen) buffer, pH 7.0) using a multichannel micropipette. The decrease in absorbance was monitored at 240 nm (Spectramax 190, Molecular Devices, Sunnyvale, CA) for 25 reads (12 s interval, total measuring time: 4 min, 17 s). The amount of peroxide decomposed was calculated using a molar coefficient of ε_240 nm, 1 cm_ = 39.4. The enzyme activity decomposing 1 µmole of hydrogen peroxide per min equals 1 unit catalase activity.

### Statistical analysis

All quantitative analyses were performed using at least three different isolates from each strain. Statistical analysis of the results was performed by applying the Mann-Whitney *U* test (two-tailed), if not noted otherwise.

## Results

### ATP content and metabolic rate in WT and mutants grisea and *PaCox17*::ble

**Figure 1:**
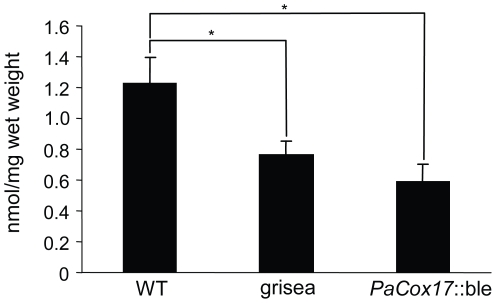
Determination of ATP content in mycelial homogenates. ATP levels were measured by a luminescence based assay. Mutants grisea and *PaCox17*::ble contain significantly less ATP than the WT. The age of the mycelia from which the homogenates were prepared is 10 d. Data represent mean ± standard error. *: p<0.05.

**Figure 2:**
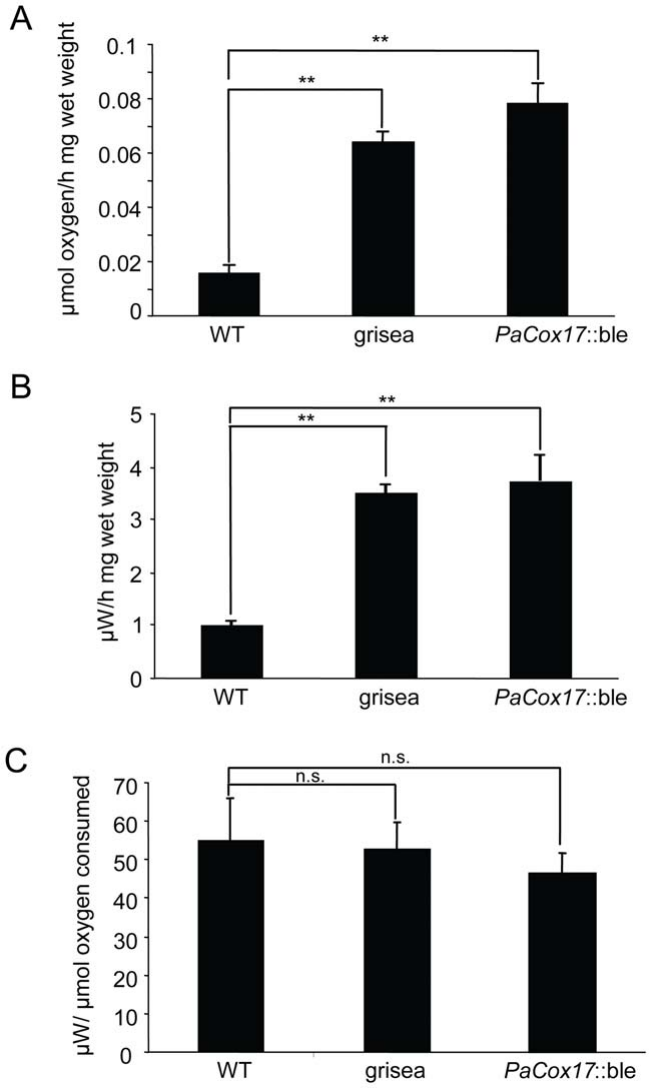
Metabolic rates of live mycelia. **A** Respirometry reveals that mutants grisea and *PaCox17*::ble are characterized by elevated oxygen consumption compared to the WT. **B** Assessment of heat production by calorimetry is also increased in the two mutants. **C** However, the mutant genotype does not influence the calorimetric/respirometric (CR) ratio. Data represent mean ± standard error. *: p<0.05; **: p<0.01; n. s.: not significant.

Long-lived *P. anserina* mutants grisea and *PaCox17*::ble are characterized by AOX-dependent respiration [13], [19]. The flow of electrons via AOX instead of COX causes fewer protons to be pumped across the inner mitochondrial membrane due to by-passing of complexes III and IV. Therefore, it is likely that ATP levels are strongly reduced in the AOX-respiring mutants. However, to our knowledge, ATP levels have not yet been measured in AOX-respiring mutants. Consequently, we set out to determine the actual effects of AOX-dependent respiration on ATP concentration in homogenates of the WT and the two long-lived mutants (Fig. 1). The data obtained from these experiments show that respiration via the AOX pathway does result in a significantly lowered ATP concentration in grisea and *PaCox17*::ble (WT: 1.24 nmol ATP/mg wet weight; grisea: 0.74 nmol ATP/mg wet weight, p<0.05 vs. WT; *PaCox17*::ble: 0.64 nmol ATP/mg wet weight, p<0.05 vs. WT). A possible strategy to compensate for ATP deficiency is an increase of catabolic rate and total metabolic rate. Indeed, these two parameters, as determined by measuring oxygen consumption and heat production of living mycelia, are significantly increased in the AOX-respiring mutants (oxygen consumption: WT: 0.016 µmol/h mg wet weight; grisea: 0.064 µmol/h mg wet weight, p<0.05 vs WT; *PaCox17*::ble: 0.076 µmol/h mg wet weight, p<0.01 vs. WT; heat production: WT: 0.91 µW/h mg wet weight; grisea: 3.38 µW/h mg wet weight, p<0.05 vs WT; *PaCox17*::ble: 3.47 µW/h mg wet weight, p<0.01 vs. WT) (Fig. 2A, B).

The mutant genotype does not influence the CR (calorimetric/respirometric) ratio, indicating that catabolic pathways are not significantly shifted towards fermentation (Fig. 2C). Collectively, these results demonstrate that increased catabolic and metabolic rates are found in mutants grisea and *PaCox17*::ble although WT levels of ATP content are not reached.

### Mitochondrial morphology and content

**Figure 3:**
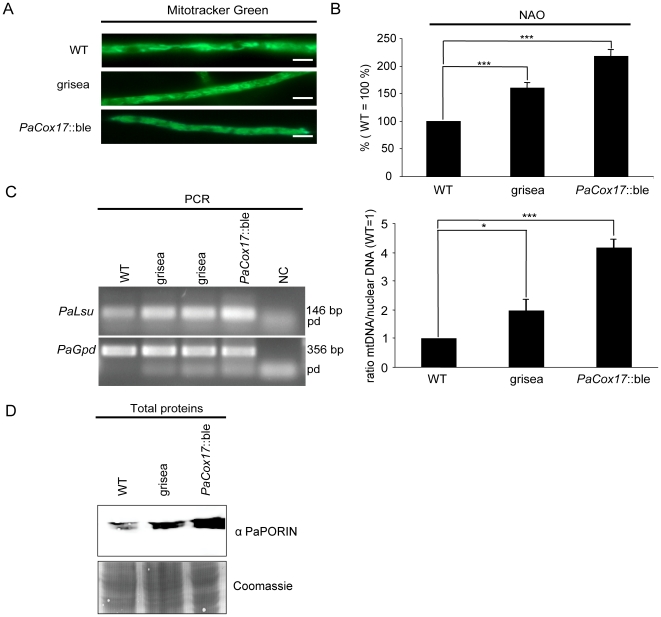
Mitochondrial content. **A** Mycelia were stained with Mitotracker Green and analysed by fluorescence microscopy. Representative hyphae are shown. Scale bar: 2 µm. **B** Protoplasts prepared from mutants grisea and *PaCox17*::ble contain significantly more mitochondria than the WT as revealed by NAO staining. Mitochondrial content in the WT was set to 100%. **C** Determination of the mtDNA/nuclear DNA ratio as a marker for mitochondrial quantity by PCR. *left*: representative 1% agarose gel showing separated *PaGpd* (nuclear DNA) and *PaLsu* (mtDNA) amplification products stained with ethidiumbromide, NC: negative control, pd: primer dimers. *right*: densitometric analysis of band intensities. The mtDNA/nuclear DNA ratio in the WT was set to 1. **D** Western blot analysis to detect PaPORIN levels in total protein extracts from the wild type strain and the two mutants. As a loading control the Coomassie-stained transfer membrane is shown. Data represent mean ± standard error. *: p<0.05; ***: p<0.001, Student's *t* test, two-tailed.

A straightforward option to increase catabolic rate and total metabolic rate is the increase of mitochondrial content in the mycelia. Are indeed more mitochondria present in grisea and *PaCox17*::ble? In order to experimentally address this question we performed a qualitative analysis by fluorescence microscopy analysis of Mitotracker Green (MTG) stained mycelia and quantified the mitochondrial content in the WT and mutants grisea and *PaCox17*::ble by NAO fluorescence analysis. MTG is a dye that is reported to stain mitochondria even when their membrane potential is very low [32]. For the study of mitochondrial content this is a desirable property. Otherwise it might be possible that a portion of cellular mitochondria are not observable. MTG-stained mitochondria of the WT and mutant grisea display a mostly filamentous morphology (Fig. 3A). Mutant grisea seems to contain shorter mitochondria than the WT. In mutant *PaCox17*::ble, filamentous mitochondria are more difficult to observe due to relatively high background fluorescence. Our results show that for quantification of mitochondrial content MTG staining analysis using whole *P. anserina* mycelia is not sufficient. As an alternative we used the dye 10-*n*-nonyl acridine orange (NAO) which binds to cardiolipin in the inner mitochondrial membrane. We found that NAO does not penetrate efficiently into intact mycelia. This effect might be due to the existence of the cell wall which can act as a barrier for certain compounds, including fluorescent dyes. Therefore we had to prepare protoplasts (i. e., fungal cells which have their cell wall removed by enzymatic digestion). When measuring NAO fluorescence as a marker for mitochondrial content, we found that mutants grisea and *PaCox17*::ble indeed contain significantly more mitochondria than the WT (WT: 100%, grisea: 160% [p<0.001 vs. WT], *PaCox17*::ble: 217% [p<0.001 vs. WT]) (Fig. 3B).

To verify these results with additional experimental methods, we measured the ratio of mtDNA/nuclear DNA as a marker of mitochondrial quantity by PCR analysis (Fig. 3C) and the level of the outer mitochondrial membrane protein PaPORIN (Fig. 3D). These experiments substantiate our NAO analysis because they show that both mutants, grisea and *PaCox17*::ble, are characterized by a significantly higher mtDNA/nuclear DNA ratio and PaPORIN levels compared to the wild type (Fig. 3C, D).

### Hydrogen peroxide production

In all organisms investigated so far, there is no clear correlation between cellular mitochondrial content and ROS production. In some studies using human fibroblasts and murine lymphoma cells, respectively, increased mitochondrial content was reported to lead to elevated ROS production [33], [34]. On the other hand, there are also analyses on various mammalian tissues reporting that a reduction of mitochondrial content leads to an increased ‘workload’ of individual mitochodria which results in a higher inner membrane potential and elevated ROS production [35]–[38]. Consequently, in order to determine the effect of increased mitochondrial content on ROS production in mutants grisea and *PaCox17*::ble we measured the production of hydrogen peroxide.

**Figure 4:**
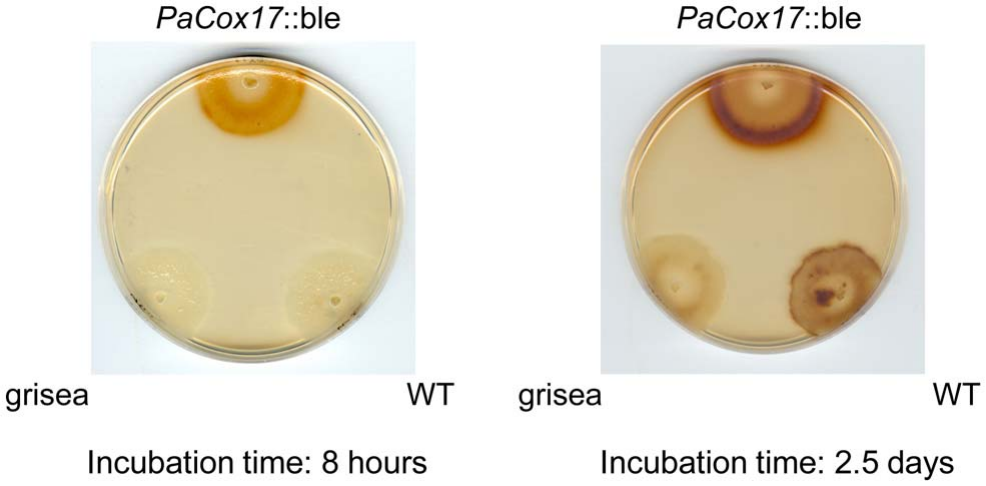
Mycelial hydrogen peroxide production. H_2_O_2_ production in WT and mutants grisea and *PaCox17*::ble as measured by mycelial DAB precipitation. While the amount of H_2_O_2_ is slightly reduced in mutant grisea compared to the WT, it is strongly increased in *PaCox17*::ble.

Hydrogen peroxide dissipated by living mycelium was measured by adding diaminobenzidine (DAB), which is oxidized by hydrogen peroxide to form a brownish precipitate. The reaction was performed on middle-aged living mycelium. We found that hydrogen peroxide production is significantly increased in *PaCox17*::ble compared to the WT (Fig. 4). We also observed that the DAB oxidation is mainly observed in the growth medium; suggesting that the mutant releases the H_2_O_2_ into the medium. Contrary to the situation in *PaCox17*::ble, hydrogen peroxide production is decreased in mutant grisea which becomes clearly evident when the DAB assay incubation time is increased to 2.5 d (Fig. 4). Taken together, our measurements on oxidative stress show that increased mitochondrial content in the mutant *PaCox17*::ble is correlated with elevated production of hydrogen peroxide but not in mutant grisea. However, it cannot be ruled out that there are sources of H_2_O_2_ production in the plasma membrane or cytoplasm beside mitochondria that are responsible for the observed effects.

### ROS scavenging

**Figure 5:**
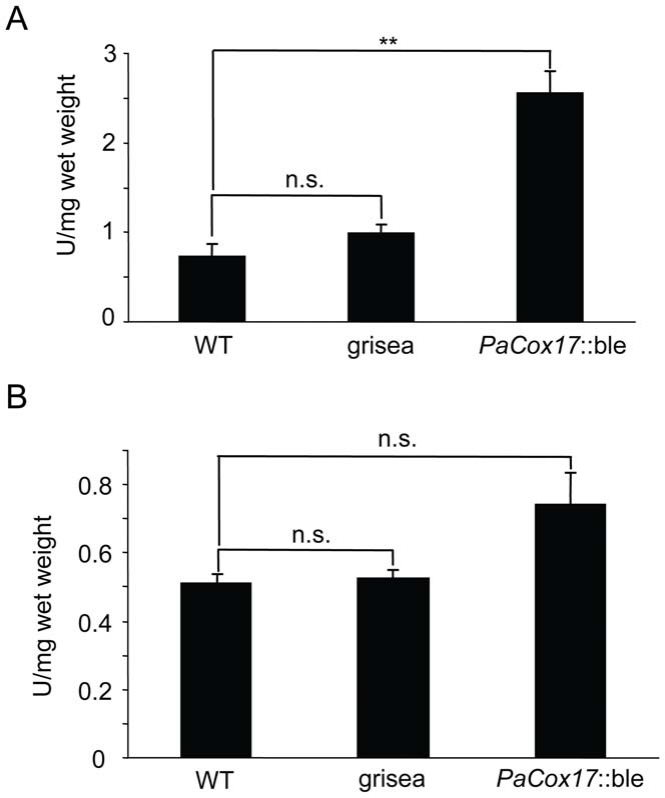
Total superoxide dismutase and catalase activity. **A** The measurement of SOD activity shows a significant increase in *PaCox17*::ble compared to the WT. **B** Catalase activity is not significantly changed between the WT and mutants grisea and *PaCox17*::ble, respectively. Data represent mean ± standard error. **: p<0.01; n. s.: not significant.

From previous investigations it is known that grisea and *PaCox17*::ble display pronounced qualitative variations in their ability to synthesize functional SODs in *in gel* activity assays [10], [13], [19]. To analyze whether the observed differences in ROS generation are due to changes in anti-oxidant capacity of the three strains, we utilized quantitative assays to measure total SOD and catalase activity. *PaCox17*::ble homogenates are characterized by a highly elevated SOD activity (Fig. 5A, 2.48 U/mg wet weight, p<0.01 vs. WT) which can be explained by the high amount of PaSOD1 present in this mutant [13]. It is certainly possible that *PaCox17*::ble produces more H_2_O_2_ due to the highly increased SOD (especially PaSOD1) activity.

Moreover, there are also no significant differences regarding catalase activity in grisea compared to the WT (Fig. 5B). Taken together, our results demonstrate that the increased mitochondrial content in the AOX-respiring mutants grisea and *PaCox17*::ble does not necessarily correlate with changes in the activity of two anti-oxidant defence systems, although total SOD activity is significantly increased in *PaCox17*::ble.

### Analysis of components of the protein quality control machinery

**Figure 6:**
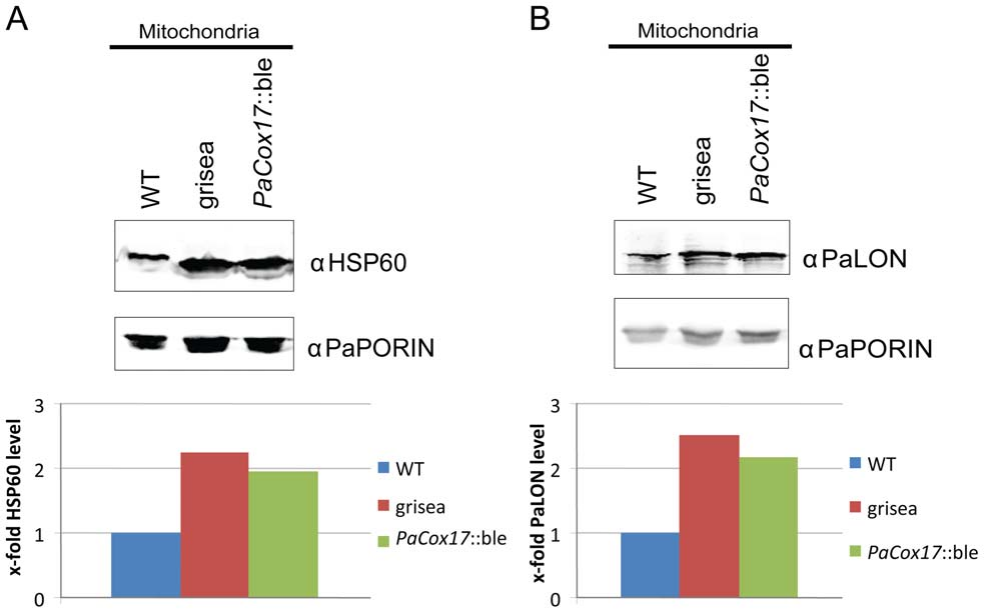
Western blot analysis of mitochondrial PaLON protease and the molecular chaperone HSP60. **A** Mitochondrial proteins in WT and mutants grisea and *PaCox17*::ble were analysed with antibodies against HSP60 after transfer to a PVDF membrane. HSP60 levels are increased in the two mutants. **B** Protein levels of LON protease (PaLON) are moderately increased in the mutants compared to the WT. Below each immunodetection a densitometric analysis of signal intensities (x-fold level compared to the WT) is shown. Intensities of the PaPORIN signals were used for normalisation. UniProt accession numbers: PaLON: B2AZ54; PaHSP60: B2B270 and PaPORIN: B2B736.

It has been demonstrated that the quality control efficiency of mitochondrial proteins (i. e., LON protease) affects the lifespan of *P. anserina* [39]. In order to investigate the levels of components of the mitochondrial quality control machinery in the long-lived mutants grisea and *PaCox17*::ble compared to the WT, we performed immunodetection analyses (Fig. 6). Mitochondrial proteins were isolated and electrophoretically separated on SDS gels. After transfer of the proteins to PVDF membranes they were decorated with antibodies against key components of quality control systems. No significant differences were found between the three strains when levels of the i-AAA protease PaIAP, CLP-Protease (PaCLPP) [40] and mitochondrial peroxiredoxin (PaPRX1) [41] were investigated (data not shown). Interestingly, both mutants show higher amounts (factor ∼2) of mitochondrial HSP60 (Fig. 6A) which has been shown to be an important factor for the proper folding of proteins imported into mitochondria of the nematode *Caenorhabditis elegans* [42]. Also the mitochondrial LON protease (PaLON) (Fig. 6B), which is involved in the efficient removal of oxidatively modified proteins [39], is present in higher amounts in the two mutants. Taken together, our experiments indicate that the two long-lived mutants contain higher protein levels of HSP60 and PaLON.

## Discussion

To gain insight into the changes of different metabolic parameters (i. e., ATP levels, oxygen consumption and heat production), hydrogen peroxide production, ROS scavenging, mitochondrial content and changes in protein levels of quality control components due to induction of alternative oxidase respiration in *P. anserina* we performed a comparative study between isolates of the WT strain and two long-lived mutants, grisea and *PaCox17*::ble. Whereas the former mutant displays residual cytochrome-c oxidase activity [43], [44], the latter is characterized by exclusive PaAOX-dependent respiration [13]. Both mutants are long-lived, although there are pronounced differences regarding the effect on lifespan. Whereas grisea is only moderately long-lived (+60% compared to the WT), *PaCox17*::ble displays a very high increase of mean life-span: forty out of sixty isolates were still alive after 320 d [13]. Like most other mutants utilising PaAOX, these strains exhibit severe phenotypical defects. *PaCox17*::ble displays an even more compromised phenotype than grisea because its growth rate is strongly decreased and it is not able to form female reproductive organs (protoperithecia) at all, resulting in sterility of the mycelium. It was assumed that the deficiencies of grisea and *PaCox17*::ble are due to insufficient generation of ATP by the PaAOX-dependent respiratory chain since two of the three sites which transfer electrons across the inner mitochondrial membrane (i. e., cytochrome-c reductase and cytochrome-c oxidase) are by-passed, resulting in a lowered proton motive force.

Our results show that mutant grisea and *PaCox17*::ble are indeed characterized by a decreased ATP content compared to the WT. Remarkably, in both mutants oxygen consumption of the mycelia is strongly increased. There does not seem to be a change in the use of metabolic pathways or mitochondrial coupling efficiency because the CR ratio, which expresses the amount of heat that is released per mol oxygen consumed, is not significantly changed compared to the WT. Furthermore, our results are in line with a former study in which the respiration rates of isolated functional mitochondria from mutant grisea were found to be significantly increased [14]. This suggests that the respiration of mycelium from mutant grisea is higher because of the added effects of a greater number of mitochondria and increased mitochondrial respiration (mitochondrial mass-specific respiration).

Interestingly, mutant grisea has been originally described as a slow-growing mutant on cornmeal agar (growth-rate: −30% compared to the wild type [16]). However, we found that freshly isolated grisea spores give rise to mycelia that grow as fast as the wild type on cornmeal agar. It is possible that more than twenty years of storage at low temperature induced physiological alterations in the grisea stock culture which lead to the formation of more mitochondria. Therefore one could argue that mutant grisea had lower levels of ATP and fewer mitochondria than it has nowadays. This hypothesis is supported by the fact that a few years ago the slow-growing mutant grisea had comparable oxygen consumption to the wild type [13]. Another explanation is that a secondary suppressor mutation in the original grisea strain appeared which leads to the wild type-like growth rate. However, it was not possible to isolate slowly growing monokaryotic progeny bearing the *grisea* gene from a genetic cross between the wild type and the mutant (unpublished results). Possibly the secondary mutation (if it exists) is linked to the locus of *grisea* and not separated by genetic crosses.

From studies in yeast it is known that there is a retrograde response pathway which signals mitochondrial dysfunction to the nucleus [45]. This induces changes in gene expression which ultimately allow for a compensation of mitochondrial defects. It is likely that a similar pathway in mutant grisea and *PaCox17*::ble results in increased mitochondrial biogenesis to achieve increased respiration and ATP production. Alternatively, an elevated down-regulation of mitochondrial degradation (e. g., by autophagy or mitophagy) in the mutants is also possible. Experimental strategies to address this question would be (i) measuring the activity of key-regulators of mitochondrial biogenesis like ‘peroxisome proliferator activated receptor gamma coactivator-1α’ (PGC-1α) or (ii) analysing mitochondrial turn-over in the WT and the PaAOX-respiring mutants used in this study.

*PaCox17*::ble produces more hydrogen peroxide than the WT in contrast to mutant grisea which produces less H_2_O_2_ (Fig. 4). It was shown that submitochondrial particles (SMPs) prepared from mutant grisea mitochondria produce less superoxide when an epinephrine based reduction assay is utilized [14]. For example, it is also known from tobacco cells that ROS production is significantly lowered in AOX-respiring cells [46]. The elevated hydrogen peroxide formation rates in *PaCox17*::ble are therefore surprising. One hypothesis to explain this finding is that mutant *PaCox17*::ble contains so many mitochondria that an elevated H_2_O_2_ production is the result. In p53^−/−^ murine lymphomas it was demonstrated that increased mitochondrial content is associated with elevated oxidative stress [33]. Oxidative stress and mitochondrial mass were also found to be positively correlated in human lung fibroblasts (MRC-5) [34]. On the other hand, it is suggested that an increase of mitochondrial content under conditions of caloric restriction leads to biogenesis of more efficient mitochondria with decreased oxidative stress as an adaptive mechanism in human skeletal muscle [47]. Therefore, the question whether the increased production of hydrogen peroxide in *PaCox17*::ble is indeed due to elevated mitochondrial content or not cannot be answered at present. Nonetheless, it is plausible that the high content of hydrogen peroxide might lead to the observed phenotypic defects (i. e., lowered growth rate, sterility) in the AOX-respiring *PaCox17*::ble mutant. How can the differences in hydrogen peroxide production between mutant grisea and *PaCox17*::ble be explained? Due to the low cellular copper levels, Cu/Zn-SOD (PaSOD1) is not active in grisea [19], [48]. By contrast, *PaCox17*::ble strongly up-regulates PaSOD1 activity [13]. The dismutation of superoxide radicals leads to the formation of hydrogen peroxide. The high levels of H_2_O_2_ in *PaCox17*::ble are likely explained by its pronounced PaSOD1 activity. Although there is a change in direction towards increased catalase levels in *PaCox17*::ble compared to the WT, these differences are not significant. It seems that an adequate up-regulation of catalase or other H_2_O_2_ detoxifying enzymes (e. g., peroxidases) is not realised in *PaCox17*::ble. Importantly, several long-lived *P. anserina* strains using AOX in addition to mutant grisea were found to produce less ROS than WT isolates (e. g., *PaCox5*::ble [11] and *cyc1-1* [49]).

If mutant *PaCox17*::ble produces more H_2_O_2_, why is it long-lived? And why does the ‘low ROS’ mutant grisea not have a higher lifespan than *PaCox17*::ble? Regarding the comparison between the WT and *PaCox17*::ble one could argue that the increase in SOD and catalase is a hormetic response to elevated hydrogen peroxide levels. A significant advantage of mutant *PaCox17*::ble over mutant grisea might be its very high activity of Cu/ZnSOD (PaSOD1) [13]. As stated above, this might also be responsible for the high levels of released H_2_O_2_. However, it is also possible that the released H_2_O_2_ is not that problematic for cellular function. Perhaps pronounced release of H_2_O_2_ is a mycelial defence mechanism in order to reduce the hydrogen peroxide concentration in the hyphae. It is possible that increased hydrogen peroxide levels also activate other stress response genes not investigated in the present study.

Contrary to the observations made in qualitative *in gel* activity assays using total protein preparations [19], [48], our results show that mutant grisea has no significant difference in total SOD activity compared to the WT. This is probably explained by the existence of a mitochondrial manganese SOD (PaSOD3) [41] which is not detected in total protein extracts but only in enriched mitochondrial protein fractions when these are analysed using *in gel* activity assays.

At present, the question whether the ‘Mitochondrial Free Radical Theory of Ageing’ [50] is to be rebutted or not is intensively discussed [51]–[53]. For example, in the nematode *Caenorhabiditis elegans* it was shown that the manipulation of genes coding for SODs leads to no major effects on lifespan, suggesting that the superoxide radical is not an important factor for lifespan determination [54]. Whatever the role of oxidative stress on the ageing process of the AOX-respiring mutants grisea and *PaCox17*::ble is, it is clear that alternative respiration and increasing mitochondrial mass to compensate for low ATP levels is no viable strategy for the realisation of ageing without functional impairments. Pathways that lead to healthy ageing in *P. anserina* and are not related to AOX-dependent respiration include (i) increase of mitochondrial fusion [26], [55], (ii) over-expression of the *O*-methyltransferase PaMTH1 to counteract deleterious metal-catalysed oxidations [23], [56] and (iii) improving mitochondrial protein quality control by over-expression of the LON protease [39].

The analysis of components of the quality control machinery by Western blot detection showed that grisea and *PaCox17*::ble seem to contain more LON protease and HSP60 than the WT. Therefore, at least in the two investigated mutants, there is a correlation between AOX dependent respiration and elevated levels of components of the molecular quality control machinery. HSP60 is known to be up-regulated during heat shock as well as during various cellular stresses [57], [58]. Interestingly, levels of HSP60 have been found to influence the levels of mitochondrial matrix proteases in a human cell line, indicating a mechanistic link in the regulation of chaperone and protease activity [59].

Mutants grisea and *PaCox17*::ble contain also moderately increased levels of the mitochondrial LON protease. Recently, experimental interventions into *PaLon* expression in *P. anserina* were demonstrated that support the importance of a functional PaLON protease in ageing [39]. Constitutive over-expression of *PaLon* resulted in transgenic strains with increased ATP-dependent serine protease activity. Notably, these strains display (i) lower levels of oxidatively modified proteins, (ii) reduced secretion of hydrogen peroxide and (iii) a higher resistance against exogenous oxidative stress. They are characterized by an extended lifespan without impairments of vital functions like growth and fertility. Collectively, these data demonstrate a beneficial effect of increasing PaLON protease abundance on stress resistance which could partially contribute to the longevity phenotype in mutants grisea and *PaCox17*::ble. However, it should be noted that *PaCox17*::ble excretes substantially more H_2_O_2_ than the wild type although it has higher PaLON levels.

Taken together, our data demonstrate significant increases of mitochondrial content in the long-lived mutants grisea and *PaCox17*::ble which both utilize PaAOX as a terminal oxidase resulting in increased oxygen consumption. We further found that mutant *PaCox17*::ble but not grisea produces more H_2_O_2_ than the WT. Although presumably not influencing lifespan, it is possible that the elevated oxidative stress leads to the phenotypic defects observed in *PaCox17*::ble. Thus, modulating pathways involving a change from COX-dependent respiration to AOX-dependent respiration does not seem to be a universal option for improving the functional lifespan (healthspan) of *P. anserina*.
